# Solving a Mystery . . . 8 Years Later

**DOI:** 10.1177/2324709617752962

**Published:** 2018-01-24

**Authors:** Hayan Jouni, Ronald S. Kuzo, Nandan S. Anavekar

**Affiliations:** 1 Mayo Clinic, Rochester, MN, USA

**Keywords:** Erdheim-Chester disease, histiocytosis, imaging, non-Langerhans cell histiocytosis

## Abstract

Erdheim-Chester disease is a rare non-Langerhans cell histiocytosis with multisystem involvement and insidious symptoms. In this article, we describe an interesting case of Erdheim-Chester disease that was eventually diagnosed 8 years after symptoms initially started.

## Case Description

A 60-year-old male was referred from an outside hospital for further evaluation of bradycardia and dyspnea on exertion. His medical history was significant for right-sided hearing loss and bilateral proptosis 8 years prior. Evaluation at that time included head magnetic resonance imaging (MRI), which revealed retrobulbar soft tissue growth but no further workup was performed. Because of worsening proptosis, he was evaluated 2 years later at which time biopsy of the retrobulbar tissue demonstrated nonspecific lymphocytic infiltrates without any histiocytes and a diagnosis of orbital pseudotumors was made. A trial of steroids was not successful in improving the proptosis, and the patient eventually underwent radiation therapy that markedly relieved his proptosis.

He has since done well until 6 weeks prior to his current presentation when he developed new-onset fatigue, night sweats, dyspnea on exertion, bradycardia, and inability to increase his heart rate beyond 90 beats per minute when he exercises. There were no other associated symptoms such as exertional chest pain, lightheadedness, syncope, vision loss, headache, bone pain, fevers, chills, or weight loss.

## Assessment

**Figure 1.**
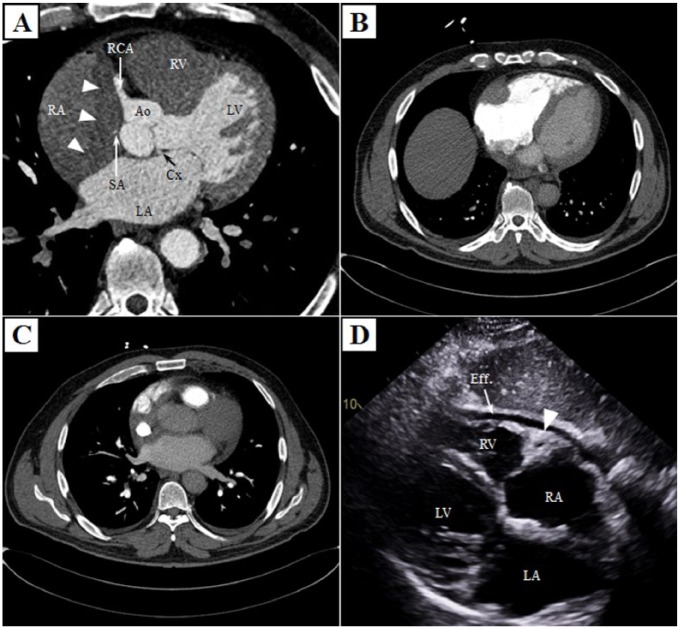
Chest computed tomography and transthoracic echocardiography. Cardiac computed tomography (with contrast timing chosen to best delineate the coronary arteries) demonstrated infiltrative soft tissue involving the right atrioventricular groove and upper portion of the interatrial septum encasing the right coronary artery and the sinoatrial node branch (A and supplementary video 1 [supplementary video is available in the online version of the article]). Chest computed tomography using the pulmonary embolism protocol allowed better visualization of the infiltrative soft tissue mass involving the right atrioventricular groove and superior aspect of the atrial septum surrounding the superior vena cava at its junction with the right atrium (B and C). Subcostal costal view of transthoracic echocardiography demonstrated a small pericardial effusion along with the soft tissue mass in the right atrioventricular groove encasing the right coronary artery (D) (Ao, aortic root; Cx, circumflex coronary artery; Eff, effusion; LA, left atrium; LV, left ventricle; RA, right atrium; RCA, right coronary artery; RV, right ventricle; SA, sinoatrial node branch).

Evaluation at the outside hospital first included a coronary computed tomography (CT) angiography, which demonstrated a soft tissue mass occupying the right atrioventricular groove encasing the right coronary artery and sinoatrial branch (Figure 1A). He also had an aberrant circumflex artery branching off the right coronary artery with a retroaortic course in addition to moderate atherosclerosis involving the left anterior descending artery. Because of shortness of breath, he subsequently underwent a pulmonary embolism CT, which better delineated the mass-like soft tissue infiltration of the right atrioventricular groove (Figure 1B) surrounding the superior vena cava at its junction with the right atrium (Figure 1C). This study also incidentally revealed soft tissue thickening surrounding both kidneys also involving the mesentery.

**Figure 2.**
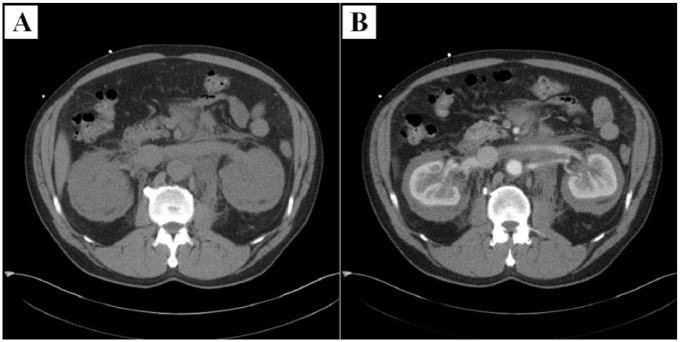
Abdomen computed tomography. Abdomen computed tomography showed an infiltrative soft tissue process in the abdomen surrounding the aorta, retroperitoneum, extending into the mesentery, and surrounding both kidneys (A). These findings mildly enhance after contrast administration (B) demonstrating the previously described “hairy kidneys” appearance in Erdheim-Chester disease.

**Figure 3.**
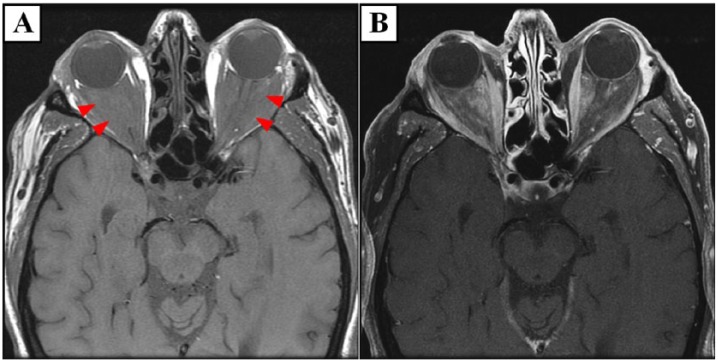
Head magnetic resonance imaging. T1-weighted head magnetic resonance images revealed intraconal abnormal soft tissue which completely fills the orbits bilaterally (A) and enhances after gadolinium administration (B).

Evaluation at our institution included an electrocardiogram that showed ectopic atrial bradycardia. A 24-hour Holter was performed demonstrating a heart rate range of 42 to 79 beats per minute with an underlying junctional rhythm as well as intermittent sinus rhythm with varying P-wave morphology. An echocardiogram was performed, which again demonstrated the soft tissue infiltration involving the right atrioventricular groove encasing the right coronary artery and a small pericardial effusion (Figure 1D and supplementary video 1). The abdominal abnormalities were better assessed with a dedicated abdomen CT as demonstrated in Figure 2. A head MRI was performed (Figure 3), which again demonstrated the previously known retrobulbar soft tissue infiltration.

**Figure 4.**
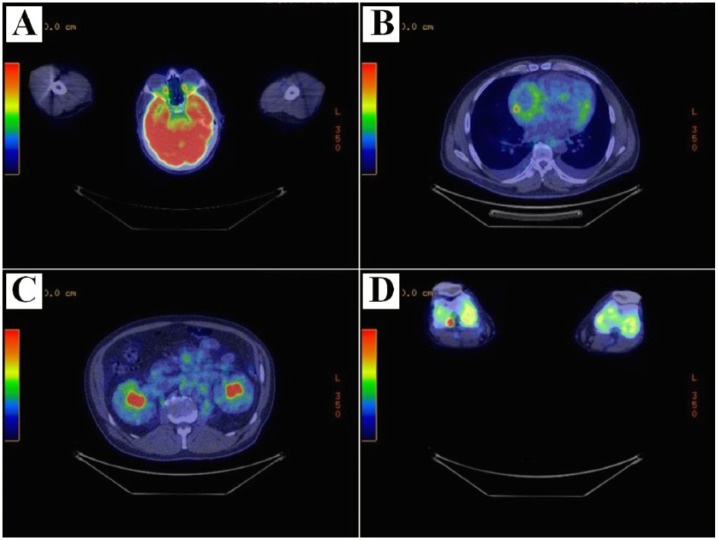
Body 18-fluorodeoxyglucose positron emission tomography showed mildly increased uptake within the orbits bilaterally (A) in addition to mild-moderate circumferential uptake about the right atrium (B). There was soft tissue thickening with mild uptake seen involving the periphery of both kidneys, central mesentery, about the aorta and inferior vena cava (C). Diffusely increased moderate activity in the bilateral distal femora and proximal tibia was seen in a pattern consistent with Erdheim-Chester disease (D).

CT and MRI findings were consistent with Erdheim-Chester disease (ECD), which prompted evaluation with hematology and biopsy of the perirenal soft tissue thickening. Biopsy demonstrated atypical dense fibrosis with chronic inflammation and lipid-laden macrophages compatible with ECD. Subsequently, an 18-fluorodeoxyglucose positron emission tomography scan was performed and demonstrated increased uptake involving the abnormal soft tissue thickening of the bilateral orbits, right atrioventricular groove, periphery of kidneys, central mesentery, and the bilateral distal femora and proximal tibia in a pattern characteristic for ECD (Figure 4). Additional immunoperoxidase studies were performed on sections of the perirenal biopsy, which were negative for IgG-4 disease and also for BRAF V600E. However, next-generation sequencing of the biopsy specimen was positive for the presence of *BRAF V600* mutation, which further supported the diagnosis of ECD. The previously obtained retro-orbital biopsy was reassessed and findings were consistent with ECD in the appropriate clinical setting. The lack of additional symptoms other than proptosis at the time of initial presentation was probably the main reason for delayed diagnosis of ECD.

## Diagnosis

The differential diagnosis for a right atrioventricular groove soft tissue mass includes lymphoma, IgG-4 disease, metastases, and ECD. Erdheim-Chester disease was first described in 1930 and is a rare non-Langerhans cell histiocytosis with multi-organ involvement and only 500 to 550 cases reported in the literature.^1^ The etiology of this disorder was unclear but is now thought to be monoclonal after discovery of *BRAF V600E* mutations in more than 50% of affected patients.^1,2^ The largest series of ECD patients in the literature included 53 patients with central nervous system involvement where the mean age was 55 years with male predominance (73%).^3^ Clinically, central nervous system manifestations and bone pain are usually present in 50% of patients. Xanthelasma, exophthalmus, or diabetes insipidus occur in approximately 20% to 30% of patients with ECD.^1^ Characteristic histopathological features include infiltration with “foamy” or lipid-laden macrophages with admixed or surrounding fibrosis. Radiographic findings include diaphyseal and metaphyseal osteosclerosis of the knees, infiltration of the perirenal fat (which results in “hairy kidneys” appearance on CT), periaortic infiltration, pericardial thickening/effusion, and right atrioventricular groove soft tissue infiltration.^1^

## Management

After confirmation of the diagnosis, the patient was started on weekly infusions of interferon-α with marked subsequent improvement of his symptoms 4 weeks after initiation of treatment. The patient’s fatigue, exertional dyspnea, and exercise intolerance all resolved. A repeat electrocardiogram demonstrated restoration of normal sinus rhythm and a repeat body positron-emission tomogram showed decreased fluorodeoxyglucose uptake as well.

Overall, management strategies for ECD are limited because of the rarity of ECD and lack of prospective randomized data. Corticosteroids may be helpful for reducing edema associated with ECD infiltration but are not considered effective as a stand-alone therapy. The mainstay of therapy for ECD is interferon-α, which demonstrated improved overall survival compared with other therapies in a case series of 53 patients with central nervous system manifestations of ECD.^3^ Anakinra, a recombinant interleukin-1 receptor antagonist, has also been shown to be effective in multiple case reports but is considered to be a second-line therapy.^1^ In ECD patients with *BRAF V600E* mutation, selective inhibition of BRAF with vemurafenib appears very promising and is currently being evaluated in prospective clinical trials.^1^

## Learning Points

- Differential diagnosis for a cardiac soft tissue mass occupying the right atrioventricular groove includes metastases, lymphoma, IgG-4 disease, and ECD.
- Diaphyseal and metaphyseal osteosclerosis of the knees with increased fluorodeoxyglucose uptake on positron emission tomography is highly suggestive of ECD.
- Interferon-α is the mainstay of therapy and gene-specific therapies are being developed.

## Supplementary Material

Supplementary material
